# Study on Reproductive Biology of *Rhododendron longipedicellatum*: A Newly Discovered and Special Threatened Plant Surviving in Limestone Habitat in Southeast Yunnan, China

**DOI:** 10.3389/fpls.2018.00033

**Published:** 2018-01-31

**Authors:** Taiqiang Li, Xiongfang Liu, Zhenghong Li, Hong Ma, Youming Wan, Xiuxian Liu, Liyong Fu

**Affiliations:** ^1^Research Institute of Resource Insects, Chinese Academy of Forestry, Kunming, China; ^2^Research Institute of Forest Resource Information Techniques, Chinese Academy of Forestry, Beijing, China

**Keywords:** limestone habitat, special threatened plant, flowering biology, reflectance spectrum, breeding system, pollination biology, *Rhododendron*

## Abstract

*Rhododendron longipedicellatum* is a narrow endemic species and a subject of urgent demand in the domestic market and overseas. Its fascinating shapes, brilliantly gilvous flowers, and unusual flowering time endow this species with extremely high ornamental value. However, only five wild populations of *R. longipedicellatum* surviving in limestone habitat have been found through elaborate field investigation, and the number of the populations decreases further or is even confronted with risk of extinction due to the damage of human activities. To enhance the protection and utilization of *R. longipedicellatum*, this study systematically investigated several important aspects of reproductive biology, including floral syndrome, pollen viability and stigma receptivity, petal color reflectance, breeding system, and pollination biology. The results demonstrated that arched styles not only create obvious herkogamy that avoide self-pollination, but also effectively reduce rain damage to the intrinsic characteristics of the stigma surface secretions, promoting the female fitness of *R. longipedicellatum* in poor weather. Pollen viability maintained a high level over the flowering period. The reflectance spectrum of petals had two peaks at wavelengths of 360 and 580 nm. Tests of OCI, P/O and artificial pollination all indicated that *R. longipedicellatum* was self-compatible and that the breeding system was mixed mating. Geitonogamy mediated by *Bombus braccatus* was the primary pollination route in the natural environment, which suggested that the breeding system of *R. longipedicellatum* might be evolving from selfing to outcrossing. The pollination vector of *R. longipedicellatum* was very specific, in that only *B. braccatus* was confirmed to deliver pollen to the stigmas. Visitation frequency was influenced by the activity rhythms and resource requirements of the different castes (i.e., sex). *B. braccatus* workers were the most effective pollinators because of higher visitation frequency and more effective contribution to fruit production, whereas the presence of *B. braccatus* males might enhance pollen flow within the population to a certain extent. Finally, these findings not only provided a reliable theoretical basis for hybridization breeding of *R. longipedicellatum* as parents, but also laid a solid foundation for further molecular biology studies to more broadly reveal the mechanisms of its endangerment in the future.

## Introduction

Reproduction is not only the most important and a relatively fragile step in the life cycle of plants but also the core of their evolutionary process. Therefore, study of the characteristics of reproductive biology of a species is indispensable in exploring the mechanisms by which it has become endangered. Numerous reports exist on the reproductive biology of *Rhododendron* L. (Escaravage et al., 1997; Mejias et al., 2002; Escaravage and Wagner, 2004; Stout, 2007; Ono et al., 2008; Epps et al., 2015; Kuswantoro, 2017). However, very few studies exist of the reproductive biology of *Rhododendron* species in the southwestern regions of China, where the diversity of *Rhododendron* is the highest; only the floral traits, flowering characteristics, pollinating insects, visitors behavior, and visitation frequency of *R. excellens, R. cyanocarpum, R. siderophyllum*, and *R. floccigerum* have been investigated (Tian, 2011; Bai et al., 2014; Ma et al., 2014b; Georgian et al., 2015).

*Rhododendron* L. is the largest genus in the family Ericaceae and one of the most widespread woody plants in the northern hemisphere. As the global distribution center of *Rhododendron*, China possesses approximately 6 subgenera and 581 species, of which 421 are endemic, and wild *Rhododendron* species are distributed in all provinces except Xinjiang and Ningxia (Chamberlain et al., 1996; Fang et al., 2005; Cai et al., 2016). As a celebrated dictum goes in European and American countries, “If it were not for the rhododendrons of China, there would be no garden in the western world.” Meanwhile, *Rhododendron* plants play important roles in maintaining biodiversity, preserving water and soil, and stabilizing the ecosystem. However, in the current century, the genetic resources of wild rhododendrons have been damaged severely due to the constant increase in human social and economic activities, and some species have become critically endangered (Ma et al., 2014a). Therefore, studies of conservation centered on reproductive biology in endangered species with high ornamental value, of which *Rhododendron longipedicellatum* is a representative, should be performed without delay.

*Rhododendron longipedicellatum* is a newly discovered and highly endangered species surviving in limestone habitat in Southeast Yunnan, during field investigations of Kunming Institute of Botany from Chinese Academy of Sciences in December 2014, is an evergreen shrub of subsect. *Pseudovireya* with a beautiful tree form, thick petal texture, and rare gilvous appearance without spots or blotch (Cai et al., 2016). Compared with other congeneric species, such as *R. sulfureum, R. wardii, R. molle*, and *R. caruolinea* growing in the United States, its appearance is more bright and pure. Surprisingly, unlike other wild rhododendrons whose flowering times occur between March and June, the natural flowering time of *R. longipedicellatum* lasts from the last 10-day period of November to the first 10-day period of February. Meanwhile, *R. longipedicellatum* is also an excellent breeding parent with important study and exploitation value. Since 2014, our study group has found five wild populations of 80–350 plants each surviving in limestone habitat in Babu Community, Malipo County, Yunnan Province, through multiple professional field investigations. The seedlings in each population are few and relatively poor in natural regeneration. The remaining partial populations have been seriously damaged by human activity; these wild resources urgently need to be protected, as their status is of grave concern. According to IUCN Red List Categories and Criteria (IUCN, 2001), *R. longipedicellatum* is a critically endangered [CR B1ab (i, iii, v)] species.

Flowers shape and color are long being thought as the result of co-evolution between flower and its pollinators (Fenster et al., 2004). Bees are considered to be pollinators of many *Rhododendron* species (Ono et al., 2008; Georgian et al., 2015), and the effective pollinating insects for *Rhododendron* species can be more specialized than previously thought, with specialization toward pollination by bumblebees (Stout, 2007). Meanwhile, some studies have indicated that yellow flowers were more preferred by bees (Robinson, 1991; Shi et al., 2008). *R. longipedicellatum* has pure yellow flowers, arched styles and unusual flowering time, we hypothesized that (1) insect visitors are various; (2) the visitation frequency of effective pollinators is higher; and (3) arched styles may be closely relate to the visit behavior of pollinating insects of this unusual plant. In order to validate the hypothesis and understand the mating system, pollination mechanism and possible strategies for reproductive assurance of *R. longipedicellatum*, this study investigates the floral syndrome, pollen viability, stigma receptivity, petal reflectance spectrum, and breeding system of *R. longipedicellatum*, as well as the types, pollination behavior and visitation frequencies of its pollinating insects. Based on the results, we further discuss the causes of its endangerment under the lens of reproductive biology; and evaluate its endangered status more comprehensively to provide a theoretical basis for its further protection and use.

## Materials and Methods

### Plant Materials and Study Sites

*Rhododendron longipedicellatum* is a multi-branched shrub 1–3.3 m tall. Its leaves are whorled, and the leaf blade is obovate to oblong-obovate, with an emarginate leaf apex. Each inflorescence possesses 1–12 flowers, with 3- to 5.5-cm-long pedicels, and 9–12 stamens uneven in length. The filaments are densely white-pubescent in the lower half but glabrous at the base and apex. The corolla is campanulate and brightly gilvous without any spots or blotch (**Figure 2A**). Most of the flowers have 5 petals, while a few have 6. *R. longipedicellatum* is distributed in limestone habitat with an altitude of approximately 1180–1320 m.

This study was conducted in the largest known population of *R. longipedicellatum* (approximately 350 mature plants) (WBL, ca. 1312 m a.s.l., 104°94′ E, 23°15′ N), while a controlled trial was conducted in the second largest population (approximately 210 mature plants) (ZWL, ca. 1270 m a.s.l., 104°93′ E, 23°16′ N) for artificial bagging pollination and observation of pollinating insects. The main accompanying plants in these habitats were limestone-loving vegetation, such as *Cyclobalanopsis glaucoides, Pistacia weinmanniifolia, Buxus sinica, R. simsii, Goldfussia pentstemonoides, Ternstroemia gymnanthera, Pieris japonica, Paphiopedilum malipoense*, and *Paphiopedilum micranthum*.

### The Biological Characteristics of *R. longipedicellatum* Flowers

#### Observation of Flowering Phenology and Floral Traits

Three inflorescences from each of 15 individuals were randomly selected. The number of flowers in each inflorescence was recorded, and the time interval from the opening of the first flower up to the wilting of the last flower in each inflorescence was observed. One flower was selected from each inflorescence, with 45 flowers in total. Each flower was labeled during the sympetalous period and observed every day until opening. The flowers were observed every 2 h on the first day of anthesis and once daily after anthesis until the corolla wilted. The duration of pollen shed, stigma secretion, and stigma bending, as well as changes in floral traits such as corolla morphology and stigma color were recorded. Subsequently, 36 flowers (from 12 individuals, 3 flowers per individual) in full bloom were selected randomly to record flower diameter, the length and width of the petals, style length, stigma length, ovary length and width, anther length, longest and shortest filament lengths, calyx lobe length and width, pedicel length, and the closest distance between anthers and stigma.

#### Detection of Pollen Viability and Stigma Receptivity

##### Detection of pollen viability

(1) Dynamic changes in pollen viability on the first day of anthesis. A total of 10 flowers (from 5 individuals, 2 flowers per individual) on the first day of anthesis were selected, and pollen was collected every 2 h from 9:00 to 21:00. The pollen from one stamen was collected and painted evenly on a lattice glass slide (Matsunami Glass, Osaka, Japan). A 5% sucrose solution containing 0.5% triphenyl tetrazolium chloride (TTC) was added to the slide. The slide was placed away from light at room temperature for 2 h. Then, the ratio of red pollen grains to all observed pollen grains in 3–5 visual fields in the center of the slide was recorded under a microscope. (2) Changes in pollen viability over different days of flowering. A total of 45 flowers (from 9 individuals, 5 flowers per individual) during the sympetalous period (mainly refers to 1∼2 days before flowering) were selected randomly. Among them, five flowers were selected, and 3–5 stamens were selected from each flower at 9:00 every day to assess pollen viability until day 9 after anthesis. Pollen viability = the number of pollen grains stained red/the total number of observed pollen grains × 100%.

##### Detection of stigma receptivity

The above 45 flowers were used as materials. Among them, five flowers were selected randomly at 11:00 every day. The stigmas were collected and immersed in a depression slide containing benzidine-hydrogen peroxide reaction solution (1% benzidine: 3% hydrogen peroxide: ddH_2_O = 4:11:22, volume ratio). A magnifier was used to observe the staining site of the stigmas after 10 min, and the change of reaction solution was recorded. The stigmas were determined to have receptivity if the reaction solution showed blue color and bubbles. Stronger receptivity was indicated by a more dramatic reaction.

#### Flower Petal Reflectance

An S2000 miniature fiber-optic spectrometer with a PX-2 pulsed xenon lamp (Ocean Optics, Dunedin, FL, United States) was used to conduct spectral measurement of 36 mature petals (from 12 different individuals, 3 flowers per individual, one petal per flower), with a wavelength range of 250–750 nm. The increments, average detection frequency and smoothness were set as 0.38 nm, 3 and 9, respectively. As preliminary testing of the reflectance pattern at 10 randomly chosen locations along the same petal did not show any variation, only one measurement per petal was used.

### The Breeding System of *R. longipedicellatum*

#### Estimation of Out-Crossing Index (OCI)

A total of 30 inflorescences (from 15 individuals, 2 inflorescences per individual) were selected randomly and their diameters were measured. Combined with the above mentioned study results of the floral syndrome, pollen viability and stigma receptivity, the hybridization index was evaluated according to the criteria of Dafni (1992).

#### Estimation of Pollen-to-Ovule (P/O) Ratio

A total of 30 flowers (from 10 individuals, 3 flowers per individual) that had bloomed but their anthers had not dehisced were selected randomly. The number of ovules in each flower was counted under a dissecting microscope. Approximately, 3–5 anthers were selected from each flower. The anthers were crushed, placed into an ethanol solution containing 0.5% methylene blue dye liquor with a metered volume of 5 ml, and then homogenized in an ultrasonic bath. Then, 10 μl of solution was extracted to count the number of pollen grains under a microscope, and this process was repeated five times to obtain the average number of pollen grains in each stamen. The total number of pollen grains in one flower = the average number of pollen grains in each stamen × the number of the stamens in the flower. The P/O ratio was estimated based on the criteria of Cruden (1977).

#### Hand Pollination Treatments

To further evaluate the breeding system of *R. longipedicellatum* scientifically, both the WBL and ZWL populations were tested with eight different types of treatments, as follows. For each treatment, 15 and 6 individuals with distances of >5 m between them were selected randomly in WBL and ZWL, respectively, and 8–10 flowers were selected from each individual at 9:00–10:00 am. The following tests were performed: (1) autonomous self-fertilization test, in which flower buds were bagged with waxed paper bags; (2) parthenogenesis test, in which flower buds were emasculated and bagged with waxed paper bags; (3) self-pollination test, in which flower buds were bagged with waxed paper bags and flowers were emasculated and artificially self-pollinated 4 days after anthesis before being rebagged; (4) geitonogamy test, in which flower buds were emasculated and bagged with waxed paper bags, and then flowers were pollinated using pollen from other flowers of the same individual 5 days after anthesis before being rebagged; (5) xenogamous pollination test, in which flower buds were emasculated and bagged with waxed paper bags, and then flowers were pollinated using pollen from different individuals at least 10 m apart 5 days after anthesis before being rebagged; (6) pollinator-mediated cross-pollination test, in which flower buds were emasculated; (7) anemophilous pollination test, in which flower buds were emasculated and bagged with mesh bags; and (8) open pollination, in which flowers were not manipulated. The flowers used in these tests were marked with tags, and fruit set was recorded at the end of February of the next year, when the seeds were mature but the fruits had not dehisced. The fruit set = the number of mature fruits/the number of flowers treated × 100%.

### The Pollination Biology of *R. longipedicellatum*

#### Collection of Pollinating Insects and Observation of Their Visiting Behavior

The pollinating insects in both the WBL and ZWL populations were observed and collected for 5 days in total during the full-bloom period (in December 2016). According to the evaluation criteria of Stout (2007), effective pollinating insects were defined as insects that not only picked up pollen but also deposited it on a receptive stigma. Thus, the effective pollinators for *R. longipedicellatum* were determined, and their visiting behavior was pictured and recorded.

#### Detection of Visitation Frequency of Effective Pollinators

Two quadrats (5 m × 5 m) were set up in each population, with more than four individuals with 30–160 flowers present in each quadrat. The visitation frequency of effective pollinators in each population was observed at 8:00–19:00 each day for 12 days in total (WBL from December 19, 2016, to December 30, 2016; ZWL from December 31, 2016, to January 11, 2017). The number of pollinators entering the quadrat to visit the flowers, the number of flowers visited and the visit time, and the number of flowers in bloom per individual were recorded daily for each quadrat. The visitation frequency was calculated as visits per flower per hour (number of flowers visited in 1 h/number of flowers in the quadrat).

#### Data Analysis

The one-sample Kolmogorov–Smirnov statistic was used to detect whether the data conformed to the normal distribution (Qu et al., 2007; Ma et al., 2012). Data with a normal distribution were subjected to pairwise comparisons using Student’s *t*-test. Significance of differences between fruit set in the hand pollination treatments was tested using a simple χ^2^-test. Two-way ANOVA was used to analyze the influence of different populations and weather conditions on visitation frequency. Both SPSS 18.0 (SPSS, Chicago, IL, United States) and SigmaPlot 12.5 (SYSTAT, Chicago, IL, United States) were used for the analysis and presentation of all data.

## Results

### Flowering Phenology and Floral Traits of *R. longipedicellatum*

The flowering time of *R. longipedicellatum* lasted from the last 10-day period of November to the first 10-day period of February in the next year, and the period from the middle 10 days of December to the middle 10 days of January was the full-bloom period. The life spans of an individual flower and inflorescence were 7.88 ± 2.09 days and 11.88 ± 2.26 days, respectively. Inflorescences were typically umbelliform or racemose and contained 1–12 flowers, with average of six (6.85 ± 2.86, *n* = 45). The stamens were 9–12 in number and uneven in length. The length of the shortest filament was 7.57–11.67 mm, significantly shorter than the 9.85–16.1 mm of the longest filament (Student’s *t*-test, *t* = 12.11; *p* < 0.001). The minimum distance between anther and stigma was 3.78 ± 1.46 mm. The primary floral morphological indexes are shown in **Table 1**.

**Table 1:** Measurements of morphological flower characters of *Rhododendron longipedicellatum*.

Characters	Sample size	Range (mm)	Mean (mm)	*SD* (mm)	CV
Flower diameter	36	28.62–45.98	36.33	4.01	0.11
Petal length	36	11.69–21.33	16.22	2.14	0.13
Petal width	36	7.65–15.84	10.73	2.02	0.19
Tube length	36	6.56–10.39	8.57	1.12	0.13
Style length	36	3.87–8.87	5.97	1.14	0.19
Stigma diameter	36	1.18–2.06	1.63	0.22	0.13
Ovary length	36	4.16–7.22	5.16	0.66	0.13
Ovary width	36	2.43–3.51	2.86	0.26	0.09
Anther length	36	2.99–5.54	3.77	0.5	0.13
Filament length (shortest)	36	7.57–11.67	9.37	1.04	0.11
Filament length (longest)	36	9.85–16.1	12.37	1.47	0.12
Calyx length	36	1.55–4.57	2.77	0.8	0.29
Calyx width	36	1.56–3.82	2.38	0.54	0.23
Pedicel length	36	30.0–55.0	41.7	7.02	0.17
Shortest distance stigma-anthers	36	0.88–6.65	3.78	1.46	0.39

*CV, coefficient of variation.*

The stamens of *R. longipedicellatum* matured first, and some anthers were mature and could release pollen during the sympetalous period. The anthers dehisced from apical pores, and adhesive silks ran through the pollen tetrads. Although the stigmas were nearly equal to the anthers in height at this stage, they were light green in color without receptivity. With the opening of the petals, the stigmas and styles bent, becoming arched in shape, which not only effectively avoided contact with the stamens but also altered the behavior of the pollinating insects. As flowering progressed, the morphology of the stigma changed from a crack-free sphere to five light cracks and then five deep cracks, while the color changed from light green to brownish red. At the end of flowering, the color of both stigmas and styles changed simultaneously to blood red (**Figure 3G**).

### Pollen Viability and Stigma Receptivity of *R. longipedicellatum*

**FIGURE 1:**
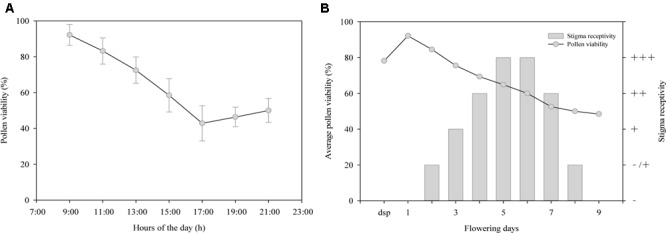
Changes of **(A,B)** pollen viability and **(B)** stigma receptivity during anthesis of *Rhododendron longipedicellatum*. dsp (during the sympetalous period, mainly refers to 1∼2 days before flowering), - stigmas have no receptivity, -/+ some stigmas have receptivity and some do not, + stigmas have receptivity, ++ stigmas have higher receptivity, +++ stigmas have the highest receptivity.

As shown in **Figure 1A**, pollen viability decreased gradually from 9:00 to 17:00 on the first day of anthesis, with the highest viability (92.18%) at 9:00 and the lowest (42.85%) at 17:00. However, the viability showed a slow increase after 17:00. As shown in **Figure 1B**, pollen viability was higher (78.20%) during the sympetalous period, but this early viability was evidently lower than that on the first day of anthesis (92.18%, Student’s *t*-test, *t* = 22.54; *p* < 0.001). Pollen viability decreased gradually as pollen grains were released from the anthers but remained relatively high (48.5%) until the end of flowering (on day 9 after anthesis).

The results of the stigma receptivity test (**Figure 1B**) showed that the stigmas were not receptive during the sympetalous period or on the first day of anthesis, as they showed no secretions or staining reaction. On days 2–6 after anthesis, stigma secretions increased and reacted more dramatically with benzidine-hydrogen peroxide reaction solution, i.e., bubbles and blue stigma staining became more obvious. On days 5–6, receptivity was the highest, during which time the stigmas were transparent with copious secretions. After that point, receptivity decreased rapidly. On day 9 after anthesis, the stigmas began to lose receptivity and wither, and then the corollas and stamens were shed.

### Petal Color Reflection of *R. longipedicellatum*

**FIGURE 2:**
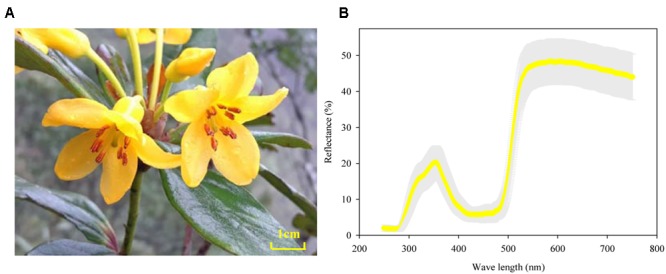
Flowers and floral reflectance spectrum of *Rhododendron longipedicellatum*. **(A)** The flower; **(B)** reflectance spectrum obtained by measuring 36 petals, with the standard deviation shown as a gray area.

The reflectance spectrums of the 36 petals measured showed a consistent distribution mode and extremely low variation between plants (gray area in **Figure 2B**). All petals had marked peaks at wavelengths of 360 and 580 nm and an obvious trough at 450 nm. Meanwhile, the gilvous flowers of *R. longipedicellatum* had a high reflection rate with a variation range of 1.80–48.6% (**Figure 2B**).

### OCI and P/O Ratio of *R. longipedicellatum*

The diameter of the inflorescences of *R. longipedicellatum* was 93.07 ± 18.53 mm (the diameter of each flower was 36.33 ± 4.01 mm), which was >6 mm and thus was scored as 3. The stamens matured first (**Figure 1B**), which was scored as 1. The arching of the style during flowering resulted in spatial isolation of stigmas and anthers, which was scored as 1. Therefore, the OCI of *R. longipedicellatum* was 5, indicating that its breeding system was partially self-compatible and outcrossing and that pollinators were needed.

The number of pollen grains, the number of ovules and the P/O ratio of *R. longipedicellatum* were 537486 ± 29414.2, 937.5 ± 77.10, and 573.3 ± 19.9, respectively. Therefore, the breeding system was facultative xenogamy.

### Hand Pollination of *R. longipedicellatum*

Both the emasculated flowers bagged with waxed paper bags and those covered with mesh bags showed no fruit set at all (**Table 2**), demonstrating that anemophilous pollination and parthenogenesis did not occur in *R. longipedicellatum*. In the flower buds bagged with waxed paper bags, the fruit set was 0 in the ZWL population and only 3.3% in the WBL population. Therefore, the possibility of autonomous self-pollination was excluded. (Five flowers in WBL population produced fruits, which might be because a small amount of pollen fell on the stigmas. However, the possibility was relatively low in the wild because the arched stigmas stick close to the base of the petals, and most mature pollen grains fell on the upper half of the petals and on the leaves.) Both sites with self-pollination had relatively higher fruit set, suggesting that *R. longipedicellatum* was generally self-compatible. The fruit set of flower buds in the emasculation and open pollination treatments were 81.3 and 92.7% in the WBL population (χ^2^ = 0.828, *p* = 0.363) and 48.3 and 58.3% in the ZWL population (χ^2^ = 0.943, *p* = 0.331), respectively, illustrating that, under natural conditions, pollinator-mediated cross-pollination was the primary pollination method for *R. longipedicellatum*. Open pollination had relatively higher fruit set than the emasculation treatment, which further indicated that the orange pollen grains of *R. longipedicellatum* might have a certain effect on attracting pollinators. The fruit set of both geitonogamous and xenogamous pollination treatments were relatively high, 88 and 90.7% in the WBL population and 83.3 and 86.7% in ZWL, respectively, which further suggested that *R. longipedicellatum* was self-compatible. Meanwhile, the fruit set in both the geitonogamous and xenogamous pollination treatments were significantly higher than that of the natural control in the ZWL population (χ^2^ = 4.433, *p* = 0.035 for geitonogamy vs. control; χ^2^ = 5.800, *p* = 0.016 for xenogamy vs. control), demonstrating that pollen limitation existed at this site.

**Table 2:** Fruit set of *Rhododendron longipedicellatum* under different pollination treatments.

Treatments	WBL			ZWL		
	Flowers	Fruits	Fruit set (%)	Flowers	Fruits	Fruit set (%)
Autonomous self-fertilization	150	5	3.3	60	0	0
Parthenogenesis	120	0	0	48	0	0
Self-pollination	150	81	67.5	60	25	41.7
Geitonogamous pollination	150	132	88	60	50	83.3
Xenogamous pollination	150	136	90.7	60	52	86.7
Pollinator-mediated cross-pollination	150	122	81.3	60	29	48.3
Anemophilous pollination	135	0	0	54	0	0
Open pollination	150	139	92.7	60	35	58.3

### Pollinating Insects and Their Visiting Behavior

A total of six kinds of pollinating insects from three orders and four families were found (**Table 3**). The visitation frequencies of *Bombus braccatus* (males and workers) and *Syrphus ribesii* were the highest, while those of *Aphid* sp. and *Apis cerana* were rare. The pollinators were similar in both populations except that no *B. braccatus* males or *A. cerana* were found in the ZWL population. The pollinators of *R. longipedicellatum* could be classified into two types according to their visiting behavior. One was disoperative insects that gnawed flower organs such as filaments, petals and stigmas, such as ants and *Aphid* sp., but this kind of insect had no contribution to pollination. The other was foraging insects that were rewarded by pollen and nectar. However, only one insect was identified as an effective pollinator, as defined by Stout (2007). Our observations suggest that pollination of *R. longipedicellatum* is nearly exclusively effected by *B. braccatus* (males and workers). Although syrphids visited at a higher frequency, they mainly picked up pollen grains from pedicels, styles and leaves, while only a few of them alighted on anthers to collect pollen directly; none of their body parts carried pollen or touched stigmas. In the WBL population, *A. cerana* visited *R. longipedicellatum* four times, but these insects only collected pollen without touching the stigmas with their bodies. Therefore, *A. cerana* was not an effective pollinator.

**Table 3:** Insects observed visiting flowers of *Rhododendron longipedicellatum*.

Order	Family	Species	Effective pollinator
Hemenoptera	Apidae	*Apis cerana*	No
		*Bombus braccatus* males	Yes
		*Bombus braccatus* workers	Yes
	Formicidae	Species	No
Deptera	Syrphidae	*Syrphus ribesii*	No
		*Episyrphus balteatus*	No
Homoptera	Aphidoidea	Species	No

**FIGURE 3:**
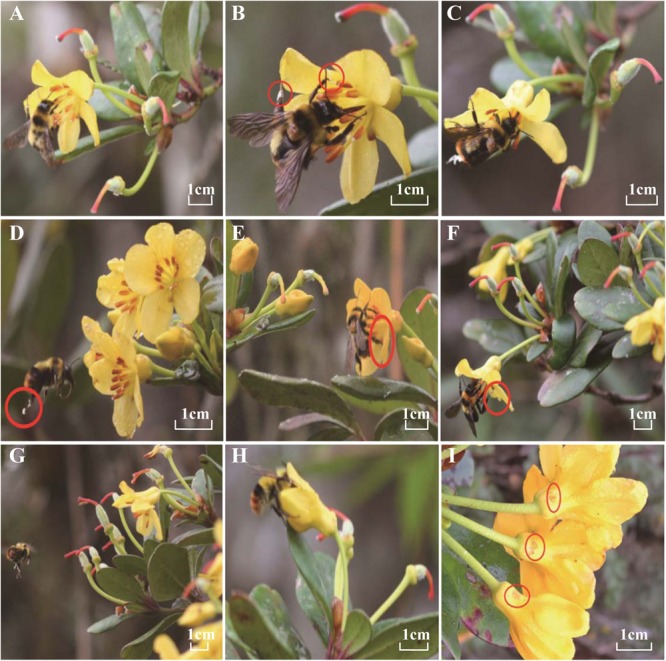
The pollination process of *Bombus braccatus*, the only effective pollinator of *Rhododendron longipedicellatum*. **(A)** *B. braccatus* alighting on the corolla. **(B)** *B. braccatus* collecting pollen and reeling linear pollen silks. **(C)** *B. braccatus* managing the pollen on the corolla. **(D)** *B. braccatus* delivering the managed pollen mass to the next flower. **(E,F)** *B. braccatus* pollinating by claws and pretarsi of metapedes. **(G)** Fruitlet after successful pollination (expanded ovary and blood red stigmas and styles). **(H)** *B. braccatus* sucking nectar on the first day of anthesis. **(I)** Punctured nectary pore. The *B. braccatus* individuals in **(A–G)** were workers, among which the one in **(F)** had one body color while the rest had another color; the *B. braccatus* in **(H)** was a male.

The style of *R. longipedicellatum* was bent into an arch, which led to herkogamy. In addition, this unusual shape was mutually adapted to the pollination habit of *B. braccatus*. The range of activity of *B. braccatus* was the space between adjacent flowers or inflorescences on one plant; *B. braccatus* moved among different plants infrequently. When collecting pollen, the whole body of *B. braccatus* alighted on one petal, with two mesopedes and one of the metapedes fixed on this petal to balance its body, whereas the pretarsi and claws of the other metapede were fixed on the edge of an adjacent petal to collect the pollen delivered from the corresponding propede (**Figure 3A**). One of the propedes was used to reel linear pollen silks from the anther pores and attach the bundled pollen to the digitus and tibias of the corresponding metapedes, while the other propede was fixed on the filament to balance the body (**Figure 3B**). When collecting nectar, *B. braccatus* fixed all feet on the petals and filaments, placed its head into the middle part of the filament, and sucked in the nectar using its long rostrum of approximately 7.3–8.7 mm (**Figure 3H**). The nectary pore selected by *B. braccatus* to collect nectar was located in the base of the second petal of the stigma in a counter-clockwise direction (**Figure 3I**). After pollen and nectar were collected, *B. braccatus* remained on the corolla to manage the pollen (**Figure 3C**). A pollen mass of approximately half the size of the stigma was managed and placed on the claws of the metapedes, while the rest of the pollen was swept into the pollen collets on the metapedes. Then, *B. braccatus* flew to the next flower (**Figure 3D**), where the sticky pollen mass was brought into light contact with the stigma by the metapedes (**Figures 3E,F**), and pollen or nectar were collected by the same method as above. During the period of maximum *B. braccatus* visitation frequency, most flowers were pollinated 1–3 times.

### Visitation Frequency of Effective Pollinators

**FIGURE 4:**
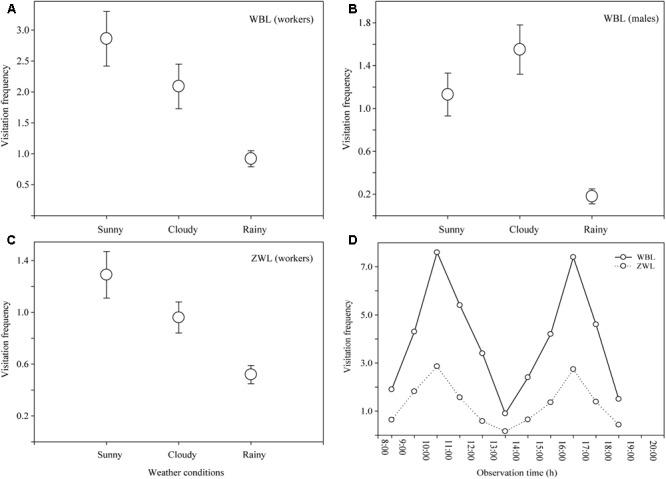
Visitation frequency (visits per flower per hour) of *Bombus braccatus* under different weather conditions in different populations. **(A)** *B. braccatus* workers (of two colors) in the WBL population. **(B)** *B. braccatus* males in the WBL population. **(C)** *B. braccatus* workers (of one color) in the ZWL population. **(D)** Curve showing the average change tendency in total visitation frequency in both populations on sunny days.

During the observational period, sunny days, cloudy days, and rainy days occurred at rates of 46, 29 and 25%, respectively. *B. braccatus* was the sole effective pollinator in both populations, but differences in the castes (i.e., sex) were present. In the WBL population, both *B. braccatus* workers of two different colors and males (28% of males and 72% of workers) occurred, whereas in the ZWL population, only *B. braccatus* workers of one color were present. The foraging time of one *B. braccatus* on the same flower and inflorescence was 11.71 ± 5.54 s (range: 4–28 s) and 33.45 ± 11.93 s (range: 9–62 s). The average visitation frequency was 3.37 ± 1.05 times flower^-1^ h^-1^ in the WBL population, which was evidently higher than the 0.95 ± 0.34 times flower^-1^ h^-1^ in the ZWL population (**Figures 4A–C**). Two-way ANOVA indicated that different populations and weather conditions had both a significant effect on the visitation frequency of *B. braccatus* (populations: *F* = 11.39, *p* = 0.002; weather conditions: *F* = 3.54, *p* = 0.042), with the interaction term between the two factors being not significant (*F* = 1.23; *p* = 0.307). In the WBL population, the visitation frequency of *B. braccatus* workers was markedly higher than that of *B. braccatus* males (Student’s *t*-test, *t* = 6.0203; *p* < 0.001), which, combined with the fact that only *B. braccatus* workers were found in the ZWL population, suggested that *B. braccatus* workers were the most effective pollinators of *R. longipedicellatum*. On sunny days, the average change tendencies in total visitation frequency of *B. braccatus* (workers and males) in both populations were almost consistent; all were in a double-peak curve with maximum visitation frequencies occurring from 10:00–11:00 and 16:00–17:00 and minimum at 13:00–14:00 due to high temperature. After 19:30, very few *B. braccatus* appeared (**Figure 4D**).

## Discussion

### Floral Traits, Pollen Viability, and Stigma Receptivity

Flower color is one of the most important ornamental characteristics for rhododendrons. At present, the breeding of flower color in rhododendrons tends to favor pure-colored flowers, especially gilvous rhododendrons. The flowering time of most wild rhododendrons falls in the period from March to June; therefore, the cultivation of *Rhododendron* varieties with different flowering times is also an important goal for the reproduction of rhododendrons (Li et al., 2008; Lan et al., 2012; Xiao et al., 2016). Rare gilvous flowers and distinctive flowering time (from the last 10-day period of November to the first 10-day period of February) will make *R. longipedicellatum* popular among rhododendrons enthusiasts.

*Rhododendron longipedicellatum* is a typical entomophilous flower. Its corolla is campanulate and marked by slight zygomorphy, and its thick petals provide a useful landing platform for pollinating insects. Many entomophilous flowers tend to adapt to insect visitation in morphological structure or floral variation, especially plants with specialized pollinating insects (Huang and Guo, 2000). In *R. longipedicellatum*, the arched styles are in close association with its specialized pollinator, *B. braccatus*. The styles bend as the petals open on the first day of anthesis, which effectively avoids body contact with the pollinator, since only the pollen-carrying feet of *B. braccatus* can deliver pollen to the stigma successfully. The arched styles can also effectively reduce rain damage to the intrinsic characteristics of the stigma surface secretions, promoting the female fitness of *R. longipedicellatum* in poor weather.

The stamens and pistils of *R. longipedicellatum* have a degree of dichogamy. When the stigma receptivity is the strongest, the pollen viability decreases, though it remains higher than 50%. Although a transient overlap period exists between male and female fertility, the arched styles visibly promote herkogamy by effectively avoiding the possibility of self-pollination. The results of a test using single, unemasculated flowers covered with waxed paper bags have also verified the above conclusion. Henceforth, when using *R. longipedicellatum* for crossbreeding, the pollen at 9:00 on the first day of anthesis are ideal if it is used as the male parent, and artificial pollination conducted on days 5–6 after anthesis will be more beneficial to seed set if it is used as the female parent.

### Flower Petal Reflectance

The reflected light of petals could also act as a signal for attracting potential pollinators (Dyer et al., 2012). The study of Ma et al. (2015) on flower color polymorphism in *R. cyanocarpum* showed that the reflectance spectrum of pink corollas had two peaks at wavelengths of 430 and 650 nm, while the white flowers had only one peak at 430 nm, and their shared pollinators (bumblebees) had a notably higher frequency of visits to pink flowers than to white flowers. The flowers of *R. longipedicellatum* are gilvous in color, which leads to high visitation frequency by *B. braccatus* on sunny days and some visits on cloudy and rainy days. The reflectance spectrum of its corollas also has two peaks, at wavelengths of 360 and 580 nm with a trough at 450 nm. The wavelengths of these peaks and troughs differ from those of *R. delavayi* and *R. cyanocarpum* (Ma et al., 2015, 2016). *B. braccatus* has three types of photoreceptor cells to distinguish substances at close range, namely, ultraviolet (short wavelength), blue (medium wavelength), and green (long wavelength) (Giurfa et al., 1996, 1997; Frey et al., 2011; Ma et al., 2014b), which have their highest sensitivities near 340, 430, and 540 nm, respectively (Peitsch et al., 1992). The spectrum data of *R. longipedicellatum* suggests that the bright yellow flowers attract *B. braccatus* to collect pollen and nectar mainly by stimulating the ultraviolet and green photoreceptors. Therefore, yellow flowers in *Rhododendron* species are markedly different from pink, red and white flowers in their reflectance spectrum, which may be one of the reasons why yellow flowers are more preferred by bees (Robinson, 1991; Shi et al., 2008).

### Breeding System

Breeding system is a manifestation of the interaction between the internal genetic mechanisms of plants and the external environment, which plays an important role in the process of evolution and feature variation in plants (Grant, 1971). Knowledge about the breeding system of a species is beneficial in making clear the characteristics of evolution and life history caused by different genetic and ecological factors, which influence allogamy and autogamy (Escaravage et al., 1997). The OCI and P/O ratio as well as the results of hand pollination treatments in *R. longipedicellatum* revealed that *R. longipedicellatum* was self-compatible and cross-fertile, and its breeding system was mixed mating. However, its arched styles effectively counteract self-pollination, indicating that the breeding system of *R. longipedicellatum* may be in the process of evolving from selfing to outcrossing. Combining these results with the visiting behavior of *B. braccatus*, an effective pollinator for *R. longipedicellatum*, this study concluded that *B. braccatus*-mediated geitonogamy might be the primary pollination method for *R. longipedicellatum* under natural conditions. There are two reasons, as follows: (1) *B. braccatus* often visits adjacent flowers and moves frequently within one inflorescence or different inflorescences on the same plant, concentrating on an area of high flower density in order to save autologous energy and substance; and (2) *R. longipedicellatum* in wild populations tends to reproduce by transverse stems, and thus, most adjacent plants are the vegetatively propagated offspring of one original plant, which further promotes the occurrence of geitonogamy. Geitonogamy is also found in multiple congeneric plants such as *R. ferrugineum* and *R. arboretum* (Escaravage and Wagner, 2004; Wheelwright et al., 2006; Stout, 2007; Ono et al., 2008).

Inbreeding is relatively common in species with small numbers of populations and individuals, narrow habitats, or small founding populations (Lee et al., 2004; Qiao et al., 2010; Tian, 2011). Inbreeding or selfing is of great significance for the preservation and reproduction of these species, but it also leads to inbreeding depression. The level of inbreeding depression can be evaluated primarily by comparing the fruit sets obtained by self-pollination and cross-pollination (Martínez-García et al., 2012). Nevertheless, the influence of inbreeding depression on offspring fitness can manifest at any stage of the life history, such as fruit set, number and germination rate of seeds, and even subsequent growth and development, as well as morphogenesis. Whether *R. longipedicellatum* has inbreeding depression and the stages at which it might manifest remain unknown. Additionally, the seedlings of *R. longipedicellatum* are rarely seen under natural conditions, which may indicate a low germination rate due to inbreeding depression, decreased seedling fitness, or an inappropriate germination environment for seeds. These questions must be further studied in the future.

### Specialized Pollinators

Bumblebees can carry large amounts of pollen securely due to their large bodies. These insects are among the most important pollinators of diverse temperate plants with poricidally dehiscent anthers, including the plants of the Ericaceae (Free, 1970; Reader, 1977; Dorr, 1981; Escaravage and Wagner, 2004). The pollinators of *Rhododendron* species are diverse, including 4 orders and 16 families (Tian, 2011). However, most studies worldwide have indicated that bumblebees were the primary effective pollinators (Mejias et al., 2002; Fenster et al., 2004; Stout, 2007; Ono et al., 2008). Of the *Rhododendron* species reported domestically, some have specialized pollinators: the effective pollinating insects are *B. festivces* and *B. richardsi* for endangered *R. cyanocarpum* (Ma et al., 2014a) and *A. cerana* for *R. excellens* (Tian, 2011). Our study demonstrated that *B. braccatus* was the only insect that not only foraged and carried pollen but also positively delivered pollen to the stigmas when visiting *R. longipedicellatum*, which supported the point of Stout (2007). In addition, compared with other *Rhododendron* species, the effective pollinating insects for *R. longipedicellatum* were more specialized.

On cloudy and rainy days when the temperature decreases to 5–14°C, *B. braccatus* still maintains a high visitation frequency (WBL, cloud: 3.63 ± 0.15 times flower^-1^h^-1^; rain: 1.09 ± 0.15; ZWL, cloud: 0.96 ± 0.12; rain: 0.52 ± 0.07), which can be attributed to its strong adaptation to an environment with low temperature and high humidity. Compared with most other pollinating insects, *B. braccatus* can forage in harsher environments (Ng and Corlett, 2000; Escaravage and Wagner, 2004).

### Visitation Frequency

During the observation of pollinating insects, no significant difference was found between the two populations based on weather conditions, but the visitation frequency of *B. braccatus* in the WBL population was notably higher than that in the ZWL population, which might be the primary cause of the high fruit set (92.7%) and lack of pollen limitation in this population under natural conditions. The difference in visitation frequency between the two populations is mainly caused by artificial factors: the ZWL population is closer to the living area of local residents where human activities are more frequent, thus leading to the persistent shrinking of the ZWL population, whereas the WBL population is more remote from the living area and free from human interference. Moreover, the difference may also be closely associated with differences in the local bumblebee fauna and forms of selection for plant reproductive traits among populations (Medel et al., 2007; Ono et al., 2008). Only a small number of *B. braccatus* workers of one body color have been found near the ZWL population, whereas a large number of *B. braccatus* males and workers of two body colors have been found near the WBL population.

On sunny days, the average visitation frequency of *B. braccatus* was 3.96 ± 0.20 times flower^-1^h^-1^ (7.63 maximum) in the WBL population, markedly higher than that of bumblebees in most other congeneric plants (Escaravage and Wagner, 2004; Ono et al., 2008). There are two primary reasons: (1) Only one plant in the area, *P. malipoense*, has an overlapping flowering time, and a microscopic examination of samples of *B. braccatus* captured in the wild has found that all pollen carried on the whole body of *B. braccatus* is the tetrad pollen characteristic of *R. longipedicellatum*. The flowers of *R. longipedicellatum* are the primary food source for *B. braccatus* in this area from the last 10-day period of November to the first 10-day period of February. (2) The success rate of pollination depends on the visiting preference of pollinating insects. Nectariferous plants or those with striking colors such as yellow or blue–violet greatly increase their attractiveness to bumblebees (Shi et al., 2008). The flowers of *R. longipedicellatum* are bright gilvous in color, which greatly increases their attraction to *B. braccatus*.

In the WBL population, the visitation frequency of *B. braccatus* workers is considerably higher than that of *B. braccatus* males, which may be related to their varied visiting habits and resource requirements. The daily routine of males consists of patrolling flights to seek unmated queens, which results in large fluctuations in their searching and foraging behaviors over the day, whereas workers constantly and intensively collect pollen and nectar for the larvae (Alcock and Alcock, 1983; Jennersten et al., 1991; Ono et al., 2008). Males are termed “vagabonds” because they have large foraging range (Williams and Dodson, 1972). In the trial, the flying distance of *B. braccatus* males was observed to be notably longer than that of *B. braccatus* workers, which increased the opportunity for xenogamy. This large range might act as a compensation mechanism for geitonogamy, decreasing the influence of inbreeding depression in *R. longipedicellatum* to some extent.

### Conservation Implications

In southwestern regions of China many species of *Rhododendron* especially those grow at low altitude face increasing threats due to anthropogenic activities and habitat degradation (Ma et al., 2014a). *R. longipedicellatum* is a newly discovered and critically endangered species, has a very limited distribution range with an altitude lower than 1400 m and only five extant limestone populations of less than 2000 individual plants in the southeast part of Yunnan province in China. Based on this situation, we propose that it should be protected for Wild Plants with Extremely Small Population program in China. Comprehensive studies on the reproductive biology are very important for small population species effective conservation and management planning. Our study of the reproductive biology of *R. longipedicellatum* indicates that the major factors that threaten the persistence of its population are ecological factors (e.g., habitat fragmentation) rather than sexual reproduction early hindered, because of the presence of high fruit set under natural conditions. Considering its habitat fragmentation and continued human disturbance, we suggest that priority for *in situ* alongside the remaining populations should be given to maintain the appropriate effective population size, especially the most severely damaged of the ZWL population, it should be regarded as a priority protection of ESU (evolutionary significant unit). Meanwhile, the local government and forest police should actively publicize to the surrounding masses to protect *R. longipedicellatum*, improve the protection awareness and participation enthusiasm of the local residents. *Ex situ* conservation and seed collection should then be carried out to provide provenance for its future recovery. Furthermore, considering the isolation of the residual populations of *R. longipedicellatum* in the wild and the limited pollination range of *B. braccatus*, corresponding measures should be undertaken to protect *R. longipedicellatum*, such as artificial enhancement of pollen flow among populations, reintroduction of plants in different populations, and artificial long-distance pollination to relieve the genetic pressure of geitonogamy on *R. longipedicellatum*. Additionally, further investigations are required to characterize the genetic diversity and structural patterns of this species within and between the extant five natural populations to provide genetic data assistant current and future conservation activities.

## Conclusion

*Rhododendron longipedicellatum* can attract pollinating insects to achieve successful reproduction with its gilvous flowers, unusual flowering time, distinctive light reflection patterns, strong pollen viability, and high output of pollen and nectar. Pollination treatments indicated that *R. longipedicellatum* was self-compatible and, cross-fertile and that the breeding system was mixed mating. The floral trait of arched styles not only leads to herkogamy and avoids the occurrence of autonomous self-fertilization at the single-flower level but also represents an adaptation to the pollination behavior of *B. braccatus*, its only effective pollinator, *B. braccatus*-mediated geitonogamy is the primary pollination mode for *R. longipedicellatum*. The interference of artificial factors greatly impacts the visitation frequency and caste distribution of *B. braccatus*, thus further influencing fruit set in *R. longipedicellatum*. Therefore, as to the protection of *R. longipedicellatum*, the protection of *B. braccatus*, is also of particular concern. Our findings support the hypothesis that the arched styles are in close association with the visit behavior of pollinating insects, and specialized pollinators (*B. braccatus*) can play a driver of evolution in *R. longipedicellatum*. These information about the reproductive biology of *R. longipedicellatum* not only has important implications for conservation and management of this special threatened plant but also is helpful for breeding system and pollination ecology studies in the multiple endangered *Rhododendron* species.

## Author Contributions

HM designed the study; ZL, HM, and LF collected the materials; TL, LF, XXL, and YW performed the experiments; TL, HM, LF, and XFL analyzed and interpreted the data; TL, XFL, and HM wrote the manuscript. All authors agree to be accountable for the final manuscript.

## Conflict of Interest Statement

The authors declare that the research was conducted in the absence of any commercial or financial relationships that could be construed as a potential conflict of interest.
